# Effects of packetization on communication dynamics in brain networks

**DOI:** 10.1162/netn_a_00360

**Published:** 2024-07-01

**Authors:** Makoto Fukushima, Kenji Leibnitz

**Affiliations:** Graduate School of Advanced Science and Engineering, Hiroshima University, Hiroshima, Japan; Center for Information and Neural Networks, National Institute of Information and Communications Technology, Osaka, Japan; Graduate School of Information Science and Technology, Osaka University, Osaka, Japan

**Keywords:** Structural brain network, Connectome, Communication model, Signal propagation, Discrete-event simulation, Packet switching

## Abstract

Computational studies in network neuroscience build models of communication dynamics in the connectome that help us understand the structure-function relationships of the brain. In these models, the dynamics of cortical signal transmission in brain networks are approximated with simple propagation strategies such as random walks and shortest path routing. Furthermore, the signal transmission dynamics in brain networks can be associated with the switching architectures of engineered communication systems (e.g., message switching and packet switching). However, it has been unclear how propagation strategies and switching architectures are related in models of brain network communication. Here, we investigate the effects of the difference between packet switching and message switching (i.e., whether signals are packetized or not) on the transmission completion time of propagation strategies when simulating signal propagation in mammalian brain networks. The results show that packetization in the connectome with hubs increases the time of the random walk strategy and does not change that of the shortest path strategy, but decreases that of more plausible strategies for brain networks that balance between communication speed and information requirements. This finding suggests an advantage of packet-switched communication in the connectome and provides new insights into modeling the communication dynamics in brain networks.

## Author Summary

Communication dynamics in brain networks have been modeled with various approximations to signaling in the connectome. These approximations differ in their assumptions about propagation strategies (random walks, shortest path routing) and switching architectures (message switching, packet switching); however, their relationships in brain network communication models have been unclear so far. Here, we link them by investigating how the difference between packet and message switching (whether signals are packetized or not) affects the transmission completion time of propagation strategies in communication simulations in the connectome. We find that packetization selectively reduces the time of physiologically plausible strategies for the connectome that balance communication speed and information requirements. This study sheds light on the utility of packet switching for modeling efficient communication in brain networks.

## INTRODUCTION

Methodological advances have enabled mapping the complete set of white matter structural connections (the connectome) in the mammalian brain (Hagmann et al., 2008; Markov et al., 2014; Oh et al., 2014; Stephan et al., 2001). Existing computational studies have investigated the communication dynamics in the connectome by modeling the flow of abstract discrete signals in the network of structural brain connections (Avena-Koenigsberger, Mišić, & Sporns, 2018; Seguin, Sporns, & Zalesky, 2023). Communication models typically rely on simple approximations of the brain dynamics, while edgewise communication metrics derived from these models explain empirical properties of functional imaging data, such as resting-state functional connectivity weights (Goñi et al., 2014; Mišić et al., 2015).

In communication models for the connectome, the dynamics of signal transmission have been approximated with various propagation strategies. The physiological plausibility of propagation strategies has been discussed in terms of communication speed and information requirements (Avena-Koenigsberger et al., 2018; Seguin, van den Heuvel, & Zalesky, 2018). The speed of interregional communication in the cortex needs to be high enough to realize complex brain functions. At the same time, the information requirements, that is, the amount of information required for a cortical signal to propagate toward its destination region (node), should be limited to the extent that no knowledge of the global network topology is used (Avena-Koenigsberger et al., 2018). Several network metrics in connectomics (Rubinov & Sporns, 2010) assume models in which communication takes place through the shortest paths between source and destination nodes; however, this requires centralized knowledge of the global network topology. Alternative models are built on the basis that nervous systems are decentralized. The simplest of such models is the random walk (Mišić, Sporns, & McIntosh, 2014; Zhou et al., 2022), where a signal randomly propagates from a node to one of its neighboring (structurally connected) nodes. The random walk (RW) and shortest path (SP) strategies are the limiting extremes in terms of communication speed and information requirements. With RW, no knowledge is required for propagation, but communication can be slow. With SP, communication is typically fast, but knowledge of the global network topology is necessary. Avena-Koenigsberger et al. (2019) proposed a biased RW strategy that balances between communication speed and information requirements.

In parallel with the discussion of propagation strategies, there is an attempt to describe communication in the connectome using an internet metaphor (Graham, 2014, 2021; Graham & Rockmore, 2011). In their model, brain network communication is realized via the propagation of packets split from each signal in a form corresponding to packet switching (Kleinrock, 1976), the switching architecture used in the internet. The physical realization of packet switching has not been established in the brain; for instance, how individual packets are correctly reassembled at the destination has remained elusive. Nevertheless, the packet-switched communication model has several potential advantages (Graham & Rockmore, 2011), including its ability to reroute network traffic to avoid congestion at hub nodes in, for example, structural brain networks (Sporns, Honey, & Kötter, 2007) and its efficiency in systems relying on temporally sparse bursts of communication through, for example, neural spiking activity (Baddeley et al., 1997, Tolhurst, Smyth, & Thompson, 2009). Graham and Rockmore (2011) contrasted packet switching with its precursors in telecommunication systems: message switching and circuit switching. In packet switching and message switching, signals are transmitted in the network toward their destination nodes under a given propagation strategy. The difference between these switching architectures is whether each signal is split into individual packets or not in transit. In circuit switching, a path of connections between source and destination nodes is first established for a signal and then used exclusively to transmit this signal.

While existing models vary in how they approximate the communication dynamics in the connectome regarding propagation strategies and switching architectures, the relationship between these two model components has not been sufficiently explored in the literature. Here, we investigate how different switching architectures affect the performance of propagation strategies in brain network communication models. We exclude circuit switching because its assumption that paths are established before transmission is incompatible with the propagation strategies we consider. Therefore, we focus on examining how splitting signals into packets changes the propagation performance. We evaluate this performance using the transmission completion time of signal propagation in mammalian brain networks modeled with discrete-event simulations. We start with the previous communication model for the connectome in Mišić, Sporns, and McIntosh (2014) for simulating message switching and modify it to simulate packet switching. As propagation strategies, we use RW, SP, and modified versions of RW that have intermediate properties between RW and SP in terms of communication speed and information requirements. We demonstrate the effects of packetization on the transmission completion time of these propagation strategies and discuss how our findings provide new insights into the physiological plausibility of brain network communication models.

## RESULTS

### Simulations of Signal Propagation in the Macaque Connectome

**Figure 1.**
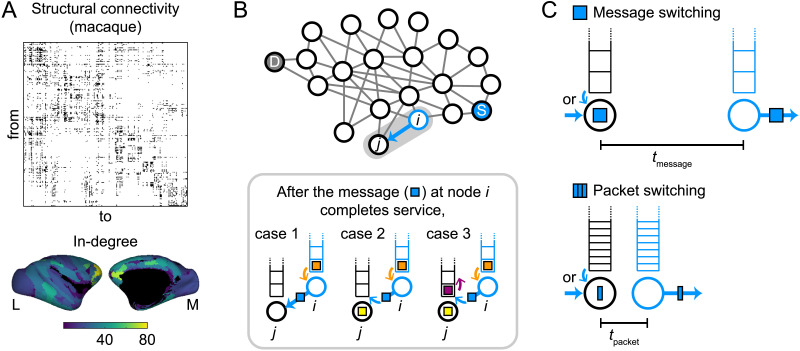
Simulation models of signal propagation in the connectome. (A) Adjacency matrix of the network of macaque structural connectivity (242 cortical nodes, 4,090 binary and directed edges) used in simulations (top) and rendering of its in-degree (the number of incoming connections to a node) on cortical surfaces (bottom left, lateral view; bottom right, medial view). The surface maps only show the in-degrees of the 187 nodes for which spatial coordinates are available (Harriger et al., 2012). (B) Schematic description of the discrete-event simulation. In simulations of signal propagation with message switching (Mišić et al., 2014; Mišić, Sporns, & McIntosh, 2014), every signal (message) was associated with a randomly generated pair of source node S and destination node D. Once the message (colored light blue) completes service at node *i*, it is transmitted to node *j* and the message (orange) in the buffer of node *i* starts service at node *i*. If there is no signal at node *j* (case 1), the light blue message immediately starts service at node *j*. If another message (yellow) is in service at node *j* (cases 2 and 3), the arriving light blue message is stored in the front of the buffer of node *j*. In case 3, where a message (purple) is in the front of the buffer of node *j*, the light blue message is stored in the front and the purple message is moved back. (C) Modification of the discrete-event simulation model to simulate signal propagation with packet switching. When a message is split into *n* equally sized packets, the service rate and the maximum number of slots in a buffer for a packet are specified as *n* times as large as those for a message. In this case, the service time for a packet (*t*_packet_) is on average that for a message (*t*_message_) divided by *n*.

We simulated the propagation of discrete signals in the connectome derived from the Collation of Connectivity data on the Macaque brain (CoCoMac) database (Harriger et al., 2012; Kötter, 2004; Modha & Singh, 2010; Stephan et al., 2001) (Figure 1A). We used the same binarized network data of structural brain connectivity as in previous studies (Mišić et al., 2014; Mišić, Sporns, & McIntosh, 2014). These studies described the communication dynamics in the connectome using a discrete-event simulation model with a queueing system (Banks & Carson, 1984) (Figure 1B). We followed their approach in our simulations of signal propagation with message switching and the RW strategy, where signals (messages) of equal length were randomly generated in time and space at source nodes in the brain network. To each message, a destination node was randomly assigned. When RW was used as the propagation strategy, the message was sequentially randomly transmitted to one of the neighboring nodes with equal probability until it arrived at its destination node. When other propagation strategies were used, the message was transmitted to a neighboring node under a given propagation strategy toward its destination node. Following the previous studies of Mišić and colleagues, nodes in the network were modeled as servers with finite buffer capacity (maximum number of slots *H* = 20; see the Supplementary Results section in the Supporting Information for the results with no buffer size limit). Once a message arrived at a node, it started service if there was no other message occupying that node, or it was stored in the buffer otherwise, forming a queue (see Figure 1B, bottom). When a message finished its service, the newest message in the buffer was to start its service (last-in-first-out queueing; Kleinrock, 1976; Banks & Carson, 1984). A message was removed from the network when it reached its destination node or when it was the oldest message in a fully occupied buffer at which another message newly arrived. We used the same arrival rate *λ* (average number of messages generated per time unit) and service rate *μ* (inverse of average service time) as in the previous studies of Mišić and colleagues (*λ* = 0.01 and *μ* = 0.02). Since the ratio *λ*/*μ* governed the system dynamics, we also report the results with decreased and increased *λ* with fixed *μ* in the Supplementary Results section.

In the discrete-event simulation model of Mišić and colleagues, an entire signal (message) was transmitted to a neighboring node at once by message switching. We modified this model to simulate signal propagation in the connectome with packet switching (Figure 1C). In the modified model, a message was divided into *n* equally sized packets. We ran simulations with *n* = 5 as the default for all packets and then also with *n* = 3 and *n* = 10 to check the reproducibility of the results (see the Supplementary Results section). Packets belonging to the same message were simultaneously generated at the source node, and each packet was independently transmitted to a neighboring node under a given propagation strategy. All packets of the same message had the same destination but could take different routes to reach it. To take the size differences of messages and packets into account, we scaled the service rate *μ* and the maximum number of slots in the buffer *H* by *n* (see Figure 1C). Further details of the simulations are described in the Materials and Methods section.

### Effects of Packetization on Transmission Completion Time

After simulating signal propagation in the connectome, we compared the transmission completion time of each propagation strategy between message switching and packet switching. We measured the completion time to transmit 100 messages or their corresponding packet sets between source and destination nodes, that is, the duration from when the first message or packet was generated in the network until the transmission of 100 messages or packet sets to their destinations was completed. We repeated the simulation and measured the completion time 100 times each for message switching and packet switching.

**Figure 2.**
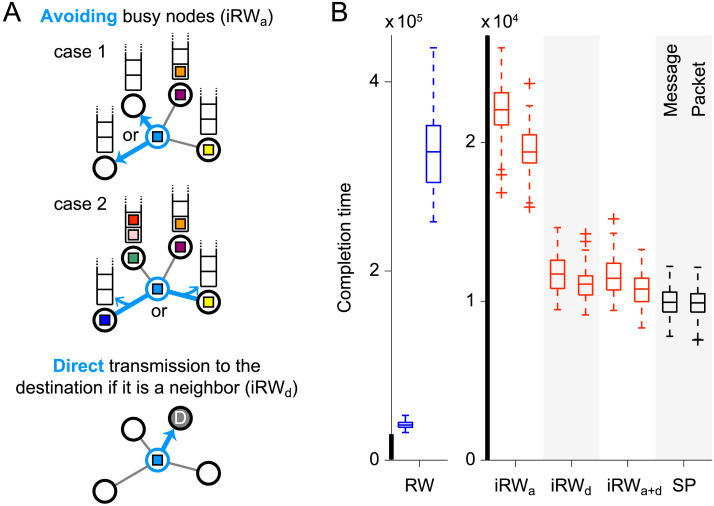
Description of iRW and results of completion time comparison for RW, iRW, and SP. (A) Schematic description of iRW. In iRW with the rule of avoiding busy nodes (iRW_a_ and iRW_a+d_), a message or packet (colored in light blue) is transmitted to a neighboring unoccupied node if it exists (case 1) or to the neighboring node with the fewest messages or packets in the buffer (case 2). In iRW with the rule of direct transmission to the neighboring destination node (iRW_d_ and iRW_a+d_), a message or packet is transmitted directly to the destination node if it is included in the set of neighboring nodes. (B) Completion time for transmitting 100 messages or packet sets in the connectome with RW, iRW_a_, iRW_d_, iRW_a+d_, and SP. The left and right columns for each propagation strategy in the boxplots show the completion times of message switching and packet switching, respectively (we ran 100 simulations to obtain the distribution of the completion time in each column). Pairs of columns for each propagation strategy are shown in red (blue) when the completion time of packet switching was shorter (longer) than that of message switching or in black when no significant differences were observed between them (*p* < 0.05, FDR corrected; see the Supporting Information for statistical details). The thick black lines in the vertical axes indicate the same range of the completion time.

We first measured the transmission completion time of RW, SP, and informed RW (iRW) that uses local information only from neighboring nodes for propagation (Figure 2A). We implemented three versions of iRW. One is a version with a rule of avoiding busy neighboring nodes in transit (iRW_a_). In this version, a message or packet is randomly transmitted to a neighboring node with no message or packet in service or, if such a node does not exist, to the neighboring node with the fewest messages or packets in the buffer. Another version is with a rule of direct transmission to the destination node if it is one of the neighboring nodes (iRW_d_). The third version is with the combination of the above two rules (iRW_a+d_).

Figure 2B shows the completion time for transmitting 100 messages or packet sets in the connectome with RW, iRW_a_, iRW_d_, iRW_a+d_, and SP. The completion time of packet switching was longer than that of message switching under RW (Mann-Whitney *U* test; *p* = 1.28 × 10^−33^, two-sided, false discovery rate (FDR) corrected by the Benjamini-Hochberg method; Cliff’s delta = 1), and the completion times of both switching architectures were similar under SP (*p* = 0.836, FDR corrected, Cliff’s delta = –0.017). By contrast, the completion time of packet switching was shorter under iRW (iRW_a_, *p* = 1.20 × 10^−20^, FDR corrected, Cliff’s delta = –0.771; iRW_d_, *p* = 5.95 × 10^−5^, FDR corrected, Cliff’s delta = –0.333; iRW_a+d_, *p* = 4.53 × 10^−7^, FDR corrected, Cliff’s delta = –0.421), indicating that splitting a message into packets improved the communication speed of iRW. All versions of iRW only need local information from neighboring nodes, but their completion times (especially those with iRW_d_ and iRW_a+d_) were much closer to the completion time of SP than that of RW. These results suggest that packetizing signals in the connectome further degrades the communication with slow strategies (e.g., RW), but conversely improves the communication with strategies that balance between communication speed and information requirements (e.g., iRW).

**Figure 3.**
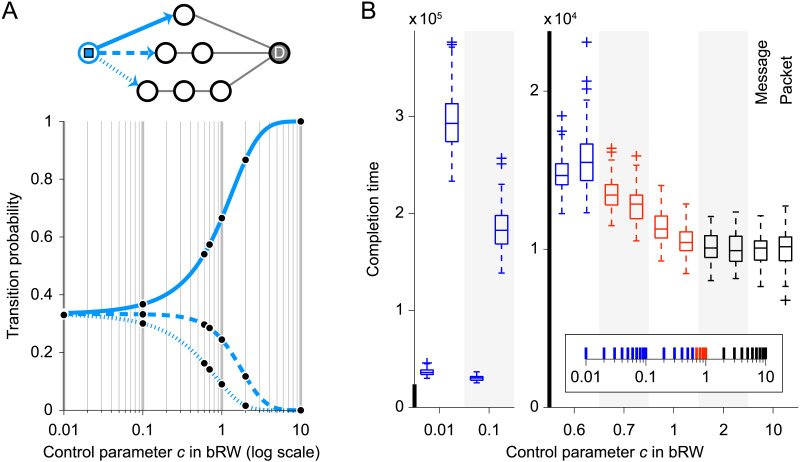
Description of bRW and results of completion time comparison for bRW. (A) Transition probability to neighboring nodes in a toy example case under bRW over the spectrum of the control parameter *c*. The transition probabilities are shown for the message or packet at the leftmost node (in the network shown above the plot) that has three neighboring nodes whose path lengths to the destination node D are one (top), two (middle), and three (bottom). The line types of the arrows toward the neighboring nodes correspond to the line types in the plot of the transition probabilities. The black dots in the plot indicate the transition probabilities at *c* for which the boxplots of the completion time are shown in panel B. (B) Completion time for transmitting 100 messages or packet sets in the connectome with bRW over the spectrum of *c*. The boxplots are colored in the same manner as in Figure 2B (blue: longer completion time of packet switching; red: shorter completion time of packet switching; black: no time difference between message switching and packet switching) (*p* < 0.05, FDR corrected for the 28 comparisons at *c* ∈ {0.01, 0.02, …, 0.09, 0.1, 0.2, …, 0.9, 1,2, …, 9, 10}). The inset located at the bottom right summarizes the statistical results at all the considered values of *c* (for their details, see the Supporting Information).

We next measured the transmission completion time of biased RW (bRW) (Avena-Koenigsberger et al., 2019) across its control parameter *c* that can shift the propagation behavior of bRW between RW and SP. Figure 3A illustrates the transition probabilities under bRW in a toy example case. When a message or packet is transmitted to one of the neighboring nodes whose path lengths to the destination node are (1) one, (2) two, and (3) three (Figure 3A, top), respectively, the transition probability to (1) is near 1/3 (one over the number of neighboring nodes; RW-like behavior) when *c* = 0.01 and near 1 (SP-like behavior) when *c* = 10 (Figure 3A, bottom). For the formal definition of the transition probability under bRW, see the Materials and Methods section. The shift of propagation behavior over the spectrum of *c* can be associated with the change in the level of availability of global information about the network structure (Avena-Koenigsberger et al., 2019). Therefore, although the shortest path lengths are required to compute the transition probabilities in the implementation, we regarded bRW with an intermediate to low range of *c* as an effective propagation strategy with respect to the information requirements.

In Figure 3B, we show the completion time for transmitting 100 messages or packet sets in the connectome under bRW across the spectrum of *c*. When *c* was relatively small, the completion time of packet switching was longer than that of message switching (e.g., *c* = 0.01, *p* = 5.52 × 10^−34^, FDR corrected, Cliff’s delta = 1) as seen in the case with RW. When *c* was relatively large on the other hand, no significant difference was observed between the completion times of message switching and packet switching (e.g., *c* = 10, *p* = 0.540, FDR corrected, Cliff’s delta = 0.060) as in the case with SP. The completion time of packet switching was shorter than that of message switching when *c* was specified within an intermediate range (e.g., *c* = 0.7, *p* = 1.53 × 10^−5^, FDR corrected, Cliff’s delta = –0.362; *c* = 1, *p* = 1.67 × 10^−9^, FDR corrected, Cliff’s delta = –0.500). In this range, the speed of communication was high and comparable to the speed under SP. These results also suggest that packetization in the connectome improves the communication with propagation strategies that balance communication speed and information requirements.

We confirmed that the results of the completion time comparisons were robust against various changes applied to the default simulation setting. We describe these changes and show the results obtained with them in the Supplementary Results section and Figures S1–S7 in the Supporting Information.

We also describe properties of individual messages and packet sets that were successfully transmitted to their destination nodes in the Supplementary Results section and Figures S8 and S9 in the Supporting Information.

### Communication Metrics in Simulations

So far, we have seen that packetization has different effects on the transmission completion times of RW, iRW, and SP and those of bRW over the range of *c*. In this subsection, we investigate its reason by computing nodewise communication metrics derived from the simulated signal propagation in the connectome. We employed communication metrics computed in the previous studies (Mišić et al., 2014; Mišić, Sporns, & McIntosh, 2014): the proportion of time that a node was busy (*utilization*) and the mean number of signals (messages or packets) at a node (*node contents*) (see the Materials and Methods section for their formal definitions). To properly compare the node contents between message switching and packet switching, we normalized the value of the node contents at each node by dividing it by the maximum number of messages or packets that each node can have.

**Figure 4.**
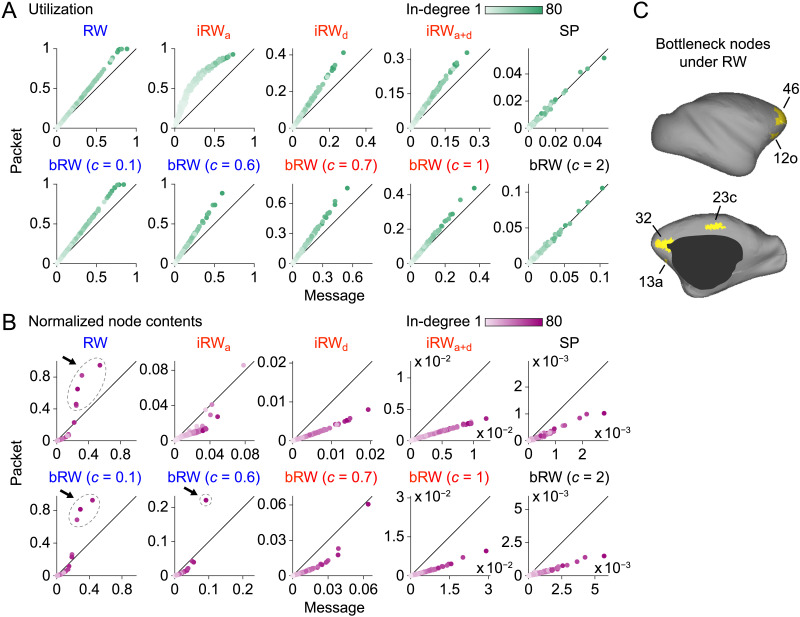
Nodewise communication metrics in the simulations of signal propagation. (A) The utilization metric for message switching and packet switching. Each dot represents a node and is colored based on the in-degree of the macaque brain network. Utilization was averaged over 100 simulation samples. Scatter plots are presented for each propagation strategy. The text color of each strategy corresponds to the color of the boxplots in Figures 2B and 3B. (B) The normalized node contents metric for message switching and packet switching. Normalized node contents were averaged over 100 simulation samples. Arrows and ellipses in the scatter plots indicate bottleneck nodes whose normalized node contents for packet switching were more than 1.5 times larger than those for message switching. (C) Spatial positions of the bottleneck nodes under RW on the cortical surface of the macaque brain.

The scatter plots in Figure 4A and B present the relations of the computed communication metrics between message switching and packet switching. The dots in the plots are colored based on the in-degree (the number of incoming connections) of the structural brain network used in the simulations (see Figure 1A). In SP and bRW with *c* = 2, utilization was almost identical between message switching and packet switching, which would explain why the completion times of both switching architectures were essentially the same under these propagation strategies. In iRW and bRW with *c* = 0.7 and 1, utilization was higher and normalized node contents were lower for packet switching in most nodes. For instance, the ratio *r* of mean utilization to mean normalized node contents under bRW with *c* = 0.1 was 0.173 for message switching and 0.799 for packet switching, indicating that packets propagated more diversely across the network compared to messages. This result suggests that packetization allows a more efficient use of the entire network, which reduces the completion time of these propagation strategies. The improvement of the ratio *r* by packetization *r*_packet_/*r*_message_ under bRW was 4.6 with *c* = 1, which was greater than 1.5 with *c* = 0.1. In RW and bRW with *c* = 0.1 and 0.6, on the other hand, normalized node contents for packet switching were more than 1.5 times larger than those for message switching at a few high in-degree nodes indicated by arrows and ellipses in Figure 4B, where some packets were dropped from the buffer. The blocking probability (Mišić, Sporns, & McIntosh, 2014) is shown in Figure S10 in the Supporting Information. These hub nodes that gathered many packets were macaque brain areas 23c (RW and bRW with *c* = 0.1) in the cingulate cortex, 12o (RW), 13a (RW), 32 (RW and bRW with *c* = 0.1), and 46 (RW and bRW with *c* = 0.1 and 0.6) in the prefrontal cortex. Figure 4C shows the positions of these areas on the cortical surface and Figure S11 in the Supporting Information displays their utilization and normalized node contents in the scatter plots. The utilization metric under RW averaged over these hub nodes for packet switching was 0.99, which means that the nodes were almost always occupied by packets. These results indicate that a few hub nodes played the role of bottlenecks in the connectome and were responsible for the longer completion time of packet-switched communication under the slow propagation strategies.

### Simulations Using Surrogate Networks

**Figure 5.**
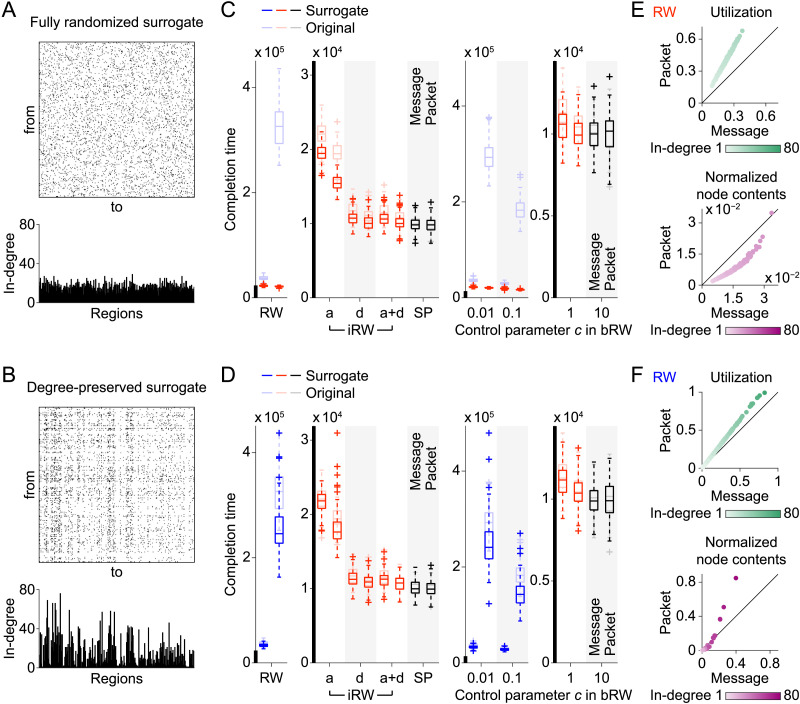
Results of the simulations using surrogate networks. (A) Adjacency matrix (top) and in-degree (bottom) of a fully randomized surrogate network. (B) Adjacency matrix (top) and in-degree (bottom) of a degree-preserved surrogate network. (C) Completion time for transmitting 100 messages or packet sets in the simulations using the fully randomized surrogate networks. (D) Completion time for transmitting 100 messages or packet sets in the simulations using the degree-preserved surrogate networks. The completion times obtained from the original brain network are also shown for reference in panels C and D. (E) Utilization (top) and normalized node contents (bottom) derived from 100 samples of the fully randomized surrogate networks. (F) Utilization (top) and normalized node contents (bottom) derived from 100 samples of the degree-preserved surrogate networks. Utilization and normalized node contents were averaged over all samples. When averaging these metrics, nodes were sorted in an ascending order of their in-degrees.

Next, we investigate how the network properties of the connectome affect the results of the completion time comparisons for message switching and packet switching. For this purpose, we simulated signal propagation in surrogate networks constructed by rewiring edges in the original structural brain network. We employed surrogate networks in which locations of edges were fully randomized (Figure 5A; no hub nodes existed) and those in which random edge rewiring was constrained to preserve the in-degree and out-degree (the numbers of incoming and outgoing connections) of nodes in the original network (Figure 5B; hub nodes remained).

Figure 5C shows the completion time for transmitting 100 messages or packet sets in the simulations using the fully randomized surrogate networks. In contrast to the results obtained from the original brain network, packetization shortened the completion time under RW and bRW with *c* = 0.1 and 0.01 (Figure 5C; for the boxplots zoomed in along the vertical axis, see Figure S12 in the Supporting Information). Bottlenecks in packet switching disappeared, where no node exhibited a utilization close to one (Figure 5E, top) or normalized node contents for packet switching more than 1.5 times larger than those of message switching (Figure 5E, bottom). On the other hand, with the degree-preserved surrogate networks, the completion time of packet switching was longer than that of message switching under the same strategies (Figure 5D), and the bottleneck nodes also remained (Figure 5F). However, the effects of packetization on the completion time were less pronounced compared to the case in the simulations using the original brain network (Figure 5D). These results suggest that hub nodes in particular, but also the entire network topology of the connectome, contribute to slowing down RW-style communication through packetization.

### Simulations in the Mouse Connectome

**Figure 6.**
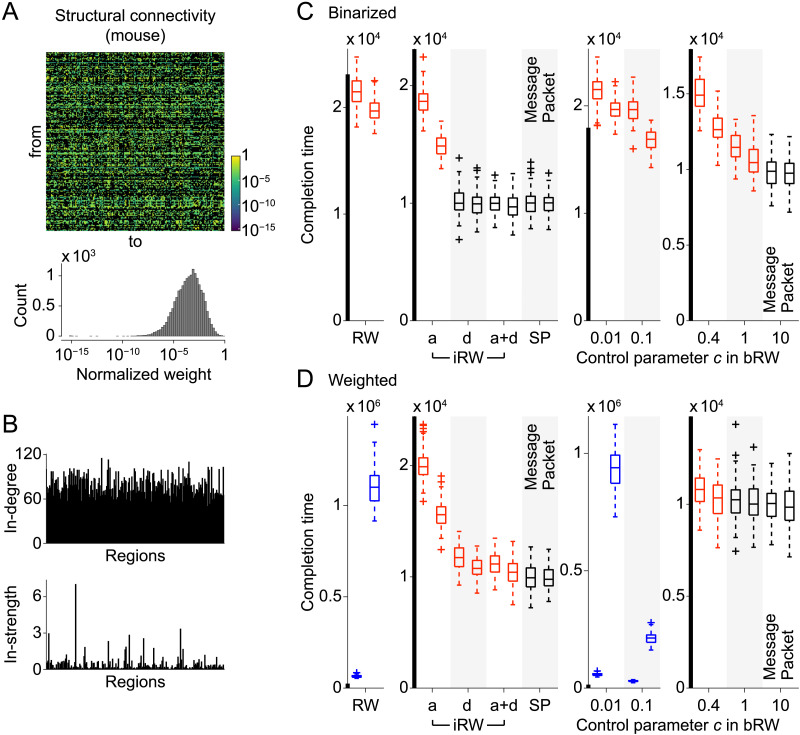
Simulations with the mouse connectome data. (A) Adjacency matrix of the network of mouse structural connectivity (213 nodes, 16,864 weighted and directed edges) used in simulations (top) and its distribution of the normalized edge weights (bottom). (B) In-degree (top) and in-strength (bottom) of the mouse brain network. (C) Completion time for transmitting 100 messages or packet sets in the simulations using the binarized mouse brain network. (D) Completion time for transmitting 100 messages or packet sets in the simulations using the weighted mouse brain network.

Finally, we demonstrate the results obtained from simulations of signal propagation in the mouse connectome (Oh et al., 2014) (Figure 6A). The number of nodes in the mouse brain network was 213, comparable to the 242 nodes in the macaque brain network used in the previous simulations. However, unlike the binarized edges in the macaque brain network, edges in the mouse brain network were weighted based on axonal projections assessed by tracer injections. We normalized the edge weights by linearly transforming them into the interval (0, 1) (Avena-Koenigsberger et al., 2019; Simas, Chavez, Rodriguez, & Diaz-Guilera, 2015). The edge weights were used to determine the transition probability and the shortest path (for more details, see Propagation Strategies in the Materials and Methods section). The edge density of the mouse brain network was 16,864/(213 × 212) = 0.373, in contrast to the density of the sparse macaque brain network 4,090/(242 × 241) = 0.070. Hub nodes were clearly visible in the plot of the nodal in-strength (the sum of weights of all incoming edges) in this network (Figure 6B, bottom), but were less evident when the edge weights were binarized (Figure 6B, top).

Figure 6C shows the completion time for transmitting 100 messages or packet sets when all edges in the mouse brain network were binarized. As seen in the results with the surrogate networks without hub nodes (Figure 5C), the completion time of packet switching was shorter than that of message switching under the slow strategies, for example, RW (*p* = 9.48 × 10^−15^, FDR corrected, Cliff’s delta = −0.644). In the binarized mouse brain network, there were no bottleneck nodes as the minimum in-degree = 50 was rather high. The completion time had no significant difference between packet switching and message switching under iRW_d_ and iRW_a+d_, where messages or packets were transmitted directly to the destination node if it was one of the neighbor nodes. This lack of difference would be due to the high edge density and the low shortest path lengths in the binarized mouse brain network (37.35% for length one, 62.49% for two, and 0.16% for three), which makes the propagation behavior of iRW_d_ and iRW_a+d_ more similar to that of SP. Figure 6D shows the results of the completion time comparisons when the weighted mouse brain network was used. As observed in the simulations using the macaque brain network with hub nodes, packetization selectively reduced the completion times under iRW (e.g., *p* = 2.69 × 10^−33^, FDR corrected, Cliff’s delta = –0.990 for iRW_a_) and bRW with intermediate *c* (e.g., *p* = 3.21 × 10^−3^, FDR corrected, Cliff’s delta = –0.262 for bRW with *c* = 0.4).

## DISCUSSION

While propagation strategies and switching architectures are key components of brain network communication models, their relationships among each other have been unclear. To address this issue, we investigated how the difference between packet switching and message switching (i.e., whether signals are packetized or not) affects the performance of propagation strategies in the connectome. By performing simulations of signal propagation in mammalian brain networks, we found that packetization in the connectome with hubs increased the transmission completion time of slow propagation strategies such as RW and did not change the time of costly SP, but decreased the time of propagation strategies that balanced between communication speed and information requirements. Nodewise communication metrics in the simulations indicate that packetization caused some high-load bottleneck nodes to appear in the connectome under the slow strategies but reduced the load on most nodes under the balanced strategies by allowing more nodes to process packetized signals. Surrogate network analysis suggests that the longer completion time through packetization in the slow strategies was not only due to the presence of high-degree hub nodes, but also due to the network topology of the connectome in the brain.

The findings in this study provide new insights into the physiological plausibility of the two major components of brain network communication models: propagation strategies and switching architectures. RW and SP are classic propagation strategies often assumed in existing models and metrics for brain network analysis (Mišić, Sporns, & McIntosh, 2014; Rubinov & Sporns, 2010). However, due to the slow communication speed of RW and the high information requirements of SP, propagation strategies that balance the speed and requirements have been considered more appropriate for use in brain network communication models (Avena-Koenigsberger et al., 2018; Seguin et al., 2018). Our finding that packetization increases the transmission completion time of RW in the connectome with hubs further supports this view. Moreover, we have shown that packet switching with balanced strategies is more advantageous than message switching for achieving faster communication in the connectome. Message switching and packet switching are both compatible with the concept of dynamic routing in the brain (Gerraty et al., 2018; Nádasdy, Hirase, Czurkó, Csicsvari, & Buzsáki, 1999; Palmigiano, Giesel, Wolf, & Battaglia, 2017). While message switching has been assumed explicitly or implicitly in many models of brain network communication (e.g., Mišić, Sporns, & McIntosh, 2014), Graham and Rockmore (2011) introduced packet switching to network neuroscience research and theoretically assessed its potential advantages over message switching for interregional communication in the brain. The present study has revealed the merit of packet switching based on empirical simulations of signal propagation in the connectome. Although there are challenges in explaining how packet-switched communication schemes are implemented in the nervous system (as noted below regarding the limitations of modeling based on packet switching), the decrease in the transmission completion time of balanced propagation strategies through packetization strengthens the value of packet switching as an element of communication models for brain networks.

A limitation of packet switching as a component of brain network communication models is that it is difficult to explain how packets in the same message are reassembled at their destination node in the connectome. Packets in the same message could be differentiated from others by communicating each message within narrow frequency bands (Arnulfo et al., 2020). However, since these packets can take different paths from their source node (Graham & Rockmore, 2011), the order in which the packets arrive at the destination can be reversed. To address this problem, an additional modeling assumption is necessary, namely that the original message is split into packets that can be reassembled in any order. One example following this assumption would be to packetize a message into different features rather than temporal fragments. An alternative solution is to model packet switching that does not allow packet overtaking. Although it reduces the flexibility of propagation, no packet overtaking can be implemented by, for example, introducing the assumption that all packets in the same message follow the path of their forerunners and replacing the last-in-first-out queueing system with first-in-first-out (Banks & Carson, 1984; Kleinrock, 1976). Even with such a model of packet switching with no packet overtaking, we confirmed that packetization reduced the transmission completion time of propagation strategies that balance communication speed and information requirements (see Figure S6 in the Supporting Information). This alternative model could alleviate concerns about explaining the physical implementation of packet reassembly in brain networks.

Another problem with the packet-switched communication model is that packetization can reduce the reliability of communication in brain networks. Errors may occur when packets are reassembled at the destination node, although we did not include such errors in the simulations. In addition, when signals are congested in the connectome, packetizing signals may make it more difficult to complete the transmission of the entire portion of individual signals to their destinations, since the probability of one of the packets being lost along the way would be greater than that of a message due to the increased number of discrete instances propagating through the network. Our simulations confirmed that slow propagation strategies such as RW caused excessive concentration of packets at bottleneck nodes, resulting in greater chances of information loss in the buffers at these nodes. The buffers that reflect the function of working memory (Funahashi, 2015; Goldman-Rakic, 1996) contribute to reliable communication, but additional mechanisms may be needed to promote the reliability of packet-switched communication. One solution is to introduce redundancy into brain network communication (Avena-Koenigsberger et al., 2017; Bettinardi et al., 2017). Duplicating signals and transmitting them through multiple paths in the connectome help improve the reliability of communication when signals collide destructively at nodes in brain networks (Hao & Graham, 2020). Duplication could be introduced into packet switching by, for example, copying several important packets to increase the chance of signal recovery at the destination node. Such a redundant propagation scheme would be necessary to make packet-switched communication more reliable even in the presence of signal congestion in the connectome.

There are also other methodological limitations to this study. First, our simulations are based on an abstract modeling approach to cortical signal propagation that is typical of many previous computational studies of brain network communication (Abdelnour, Voss, & Raj, 2014; Avena-Koenigsberger et al., 2018, 2019; Crofts & Higham, 2009; Goñi et al., 2014; Mišić et al., 2015; Mišić et al., 2014; Mišić, Sporns, & McIntosh, 2014; Seguin, Sporns, & Zalesky, 2023; Seguin et al., 2018). We focused on this approach because it allows us to trace the propagation of individual signaling units in the connectome. However, the results should be interpreted with the caveat that the models used here are simpler than the more realistic biophysical models of neuronal population dynamics that can reproduce ongoing large-scale brain activities (Breakspear, 2017; Cabral, Hugues, Sporns, & Deco, 2011; Deco et al., 2017; Deco & Jirsa, 2012; Fukushima & Sporns, 2018, 2020; Honey, Kötter, Breakspear, & Sporns, 2007; Honey et al., 2009; Pope, Fukushima, Betzel, & Sporns, 2021). In addition, all nodes and edges were treated equally in the models, despite the fact that brain regions vary in size and their structural connections vary in length and width. The models were configured in this way to avoid making even stronger assumptions about how these variations affect the dynamics of communication in brain networks. Second, only a limited number of propagation strategies were used in the simulations. A single propagation strategy was assumed for all communication processes in an individual simulation sample, whereas it is possible that neural systems may combine aspects of multiple propagation strategies (Avena-Koenigsberger et al., 2018; Betzel, Faskowitz, Mišić, Sporns, & Seguin, 2022; Z. Q. Liu et al., 2022; Vázquez-Rodríguez et al., 2019). Navigation (Seguin et al., 2018) and broadcasting (Mišić et al., 2015) strategies were also not investigated because navigation requires the location information of nodes, which is missing for several nodes in the macaque brain network used in this study, and broadcasting multiplies signals over time, making it difficult to compare results with those obtained from conventional propagation strategies (e.g., RW and SP). Third, the surrogate network analysis in the present study can be further elaborated to explore which architectural features of the connectome underlie the results. We could find such features by using topology-constrained surrogates that preserve key network features of the connectome (e.g., community structure) (Fukushima & Sporns, 2020). Furthermore, we could assess the contribution of spatial embedding of the connectome by introducing the time delays at edges into the simulations and using geometry-constrained surrogates that preserve the relationship between connectivity weights and lengths (Roberts et al., 2016).

The current study is a first step toward a comprehensive assessment of the physiological plausibility of propagation strategies and switching architectures in brain network communication models. We have demonstrated the advantage of packet switching for communication in brain networks under propagation strategies that balance communication speed and information requirements; however, this finding only indirectly supports the plausibility of this switching architecture. For more direct evidence, it would be necessary to include evaluations based on empirical functional data (Seguin et al., 2023; Seguin, Tian, & Zalesky, 2020). A promising approach for such evaluation is to compute the similarity between edgewise communication metrics quantifying the frequency of simulated signal transmission (Mišić, Sporns, & McIntosh, 2014) and the weights of empirical resting-state functional connectivity (Damoiseaux et al., 2006; Fox et al., 2005; Yeo et al., 2011). Computing this similarity, which quantifies the extent to which a communication model can explain empirical functional interactions in the brain, may allow us to evaluate the plausibility of a given combination of propagation strategy and switching architecture.

## MATERIALS AND METHODS

### Connectome Data

The macaque connectome data were derived from the CoCoMac database (Kötter, 2004; Stephan et al., 2001) that provides white matter structural connectivity information of the macaque brain reported in existing tract tracing studies. Connectivity information was initially collected by querying this database in Modha and Singh (2010), later refined to obtain a fully connected brain network (Harriger et al., 2012), and used in simulations of signal propagation in Mišić, Sporns, and McIntosh (2014) and Mišić et al. (2014). This structural brain network was comprised of 242 cortical regions (nodes) and 4,090 directed connections (edges) represented in binary format (connection present = 1 and absent = 0) and contained no self-connections (see Figure 1A). The adjacency matrix of the network and a list of the region names are available in the Supporting Information.

The mouse connectome data were downloaded from the supplementary information of Oh et al. (2014) (’W_ipsi’ in 41586_2014_BFnature13186_MOESM71_ESM.xlsx). This brain network had 213 nodes and 16,864 directed edges (without self-connections) that were weighted based on axonal projections assessed by tracer injections (Figure 6A). The edge weights of the mouse brain network were then linearly transformed to obtain normalized edge weights in the interval (0, 1) as in Simas et al. (2015) and Avena-Koenigsberger et al. (2019).

### Discrete-Event Simulation and Switching Architectures

Signal propagation in the connectome was simulated using discrete-event simulation techniques (Banks & Carson, 1984). In the simulations with message switching, individual signals (messages) were randomly generated in the brain network as a Poisson process with exponentially distributed interarrival times (arrival rate *λ* = 0.01 in the default setting) as in Mišić, Sporns, and McIntosh (2014) and Mišić et al. (2014). Poisson arrivals represent statistical fluctuations of the sensory environment (Barlow, 1956; McGill, 1967) and also reflect real arrival times in the internet. To each generated message, a pair of source and destination nodes were randomly assigned. A message was transmitted to one of the neighboring nodes under a given propagation strategy until it reached its destination node. After reaching the destination node, the message was removed from the network. The time that a message spent at each node (service time) was exponentially distributed (service rate *μ* = 0.02) as in the previous studies of Mišić and colleagues. The ratio of the arrival rate to the service rate (*λ*/*μ*) governed the dynamics of the whole system and was specified so that the number of messages in the network did not monotonically increase during the simulations. If a message arrived at a node that was already occupied by another message, the arriving message was placed in a buffer and formed a queue. Queueing was used to ensure that the dynamics of the messages were interdependent (Y. Liu, 1996). Messages entered the node on a last-in-first-out basis (Banks & Carson, 1984; Kleinrock, 1976), and a maximum buffer size was imposed (*H* = 20) in the default setting as in the previous studies of Mišić and colleagues. In this case, a message arriving at a fully occupied buffer caused the oldest message in the queue to be ejected and removed from the network. The last-in-first-out queuing rule models the natural temporal decay of biological signals (Mišić et al., 2014) (cf. first-in-first-out queuing assumes no decay). The finite buffer size was used to model imperfect signal transmission (Faisal, Selen, & Wolpert, 2008). The state of the system was updated at nonuniform intervals because of the presence of stochastic variables in interarrival times and service times. Figure 1B shows a schematic description of the discrete-event simulation with message switching.

In the simulations with packet switching, packets split from the entire message were transmitted across the network. A message was divided into *n* = 5 equally sized packets in the default setting. A set of *n* packets was simultaneously generated at a source node and transmitted to neighboring nodes under a given propagation strategy toward their common destination node. Packets in the same message individually propagated over the network in the default setting, such that they could take different routes and arrive at the destination node in a different order with different propagation delays. The service rate and the maximum number of slots in a buffer for a packet were specified as *nμ* = 0.1 and *nH* = 100, respectively (Figure 1C).

### Comparison of Transmission Completion Time

The transmission completion time of each propagation strategy was compared between message switching and packet switching to investigate the effects of packetization. We measured the completion time in simulations to transmit 100 messages or their corresponding packet sets to their respective destination nodes in the connectome. The completion time of message switching was defined as the duration from when the first message was generated at a source node until when 100 messages in total arrived at their respective destination nodes. The completion time of packet switching was the duration from when the first set of packets was generated at a source node until when all *n* packets in 100 packet sets arrived at their respective destination nodes. We ran 100 simulations for message switching and for packet switching. Using these simulation samples, we assessed whether the completion time of packet switching was shorter or longer than that of message switching for each of the propagation strategies described below.

### Propagation Strategies

We compared the transmission completion time between message switching and packet switching under the following strategies of signal propagation in the connectome: random walk (RW), shortest path (SP), and informed and biased versions of RW (iRW and bRW) that have intermediate properties between RW and SP in terms of communication speed and information requirements.

In RW, a message or packet was randomly transmitted to one of the neighboring nodes of the current node with equal probability for binarized networks. When weighted networks were used, the transition probability was linearly weighted according to the normalized edge weights. In this strategy, no global information about the network structure was required for signal propagation. RW was used in the previous implementation of discrete-event simulations in Mišić, Sporns, and McIntosh (2014) and Mišić et al. (2014).

In SP, a message or packet was transmitted along the shortest path between the source and destination nodes. For weighted networks, the shortest path was determined based on the distances computed by taking the logarithm of the inverse of the normalized edge weights (Avena-Koenigsberger et al., 2019), where the path length was the total distance of the edges along the path. If multiple shortest paths existed, a message or packet was randomly transmitted to one of the neighboring nodes along a shortest path. For signal propagation with this strategy, full connectivity information of the network was necessary to determine the shortest path. Propagation with SP has been assumed in the definitions of several network metrics, for example, global efficiency (Latora & Marchiori, 2001; Rubinov & Sporns, 2010).

In contrast to SP, iRW only uses local information from neighboring nodes. We implemented three different versions of iRW (Figure 2A): (1) iRW with a rule of avoiding busy nodes (iRW_a_), (2) iRW with a rule of direct transmission to the destination node if it is one of the neighboring nodes (iRW_d_), and (3) iRW with both of these rules combined (iRW_a+d_). In iRW_a_ and iRW_a+d_, a message or packet was transmitted to an unoccupied neighboring node if it existed. Otherwise, a message or packet was transmitted to the neighboring node with the shortest queue length in its buffer. If there were multiple such candidate nodes to transmit a message or packet, one of them was randomly selected in the manner of RW. In iRW_d_ and iRW_a+d_, a message or packet was transmitted directly to the destination node if it was in the set of neighboring nodes to the current node.

In bRW (Avena-Koenigsberger et al., 2019), the propagation behavior changes between that of RW and SP through the control parameter *c* in *p*_*ijD*_ = exp(−(*c*(*d*_*ij*_ + *g*_*jD*_) + *d*_*ij*_))/*Z*_*iD*_, where *p*_*ijD*_ is the probability that a message or packet was transmitted from the current node *i* to one of its neighboring nodes *j* when *D* is the destination node of the message or packet, *d*_*ij*_ is the distance of the edge connecting nodes *i* and *j*, *g*_*jD*_ is the shortest path length between node *j* and destination *D*, and *Z*_*iD*_ = ∑_*j*_ exp(−(*c*(*d*_*ij*_ + *g*_*jD*_) + *d*_*ij*_)) is a normalization factor. When the network was binarized, all edges were assigned the same distance of 1; otherwise, *d*_*ij*_ = −log(1/*w*_*ij*_), where *w*_*ij*_ is the normalized edge weight between nodes *i* and *j*. The term *d*_*ij*_ + *g*_*jD*_ corresponds to the minimum total distance between node *i* and destination *D* via neighboring node *j*. The control parameter *c* can change the extent to which the global information about the network structure is available for signal propagation (see Figure 3A for how *c* changes the transition probability in a toy example case). If *c* = 0, the transition probability *p*_*ijD*_ is reduced to that of RW as *p*_*ijD*_ becomes the same across all neighboring nodes for binarized networks or the transition probabilities depends only on the normalized edge weights for weighted networks. If *c* → ∞, *p*_*ijD*_ is reduced to that of SP as *p*_*ijD*_ → 1 at a neighboring node *j* for which *d*_*ij*_ + *g*_*jD*_ is the minimum. Furthermore, if there are two or more such neighboring nodes due to the existence of multiple shortest paths, the transition probabilities are computed as in SP.

### Communication Metrics

From the simulated signal propagation in the connectome, we computed two nodewise communication metrics: the proportion of the time that a node was busy (*utilization*) and the mean number of messages or packets at a node (*node contents*) as in Mišić, Sporns, and McIntosh (2014) and Mišić et al. (2014). These metrics were derived from the following simulation variables for node *i* at time *t*: the server contents *s*_*i*_(*t*) ∈ {0, 1}, which represents whether there is a message or packet currently in service (*s*_*i*_(*t*) = 1) or not (*s*_*i*_(*t*) = 0), and the queue length *q*_*i*_(*t*) ∈ {0, …, *H*} (message switching) or {0, …, *nH*} (packet switching), which represents the number of messages or packets in the buffer. The utilization of node *i* was defined as the proportion of simulation time having *s*_*i*_(*t*) = 1. The node contents were defined as the sum of the server and queue contents, *n*_*i*_(*t*) = *s*_*i*_(*t*) + *q*_*i*_(*t*). The value of the node contents at node *i* was normalized by dividing it by the maximum value that *n*_*i*_(*t*) can take (1 + *H* for message switching or 1 + *nH* for packet switching).

### Surrogate Networks

In the surrogate data analysis, we constructed two types of surrogate networks by rewiring the edges in the original structural brain network. We considered (1) fully randomized surrogate networks in which edges were randomly rewired with no constraint and (2) degree-preserved surrogate networks in which edges were rewired while preserving the sequences of the in-degree and out-degree of nodes in the original brain network. We generated 100 different realizations of the fully randomized or degree-preserved surrogate networks to obtain the distributions of the completion time in Figure 5C or D. The same sets of the 100 surrogate networks were used in the simulations of signal propagation for each combination of the propagation strategies and switching architectures.

## SUPPORTING INFORMATION

Supporting information for this article is available at https://doi.org/10.1162/netn_a_00360. The source data of all figures and the code for generating all simulation results are available in the Supporting Information.

## AUTHOR CONTRIBUTIONS

Makoto Fukushima: Conceptualization; Data curation; Formal analysis; Funding acquisition; Investigation; Methodology; Project administration; Resources; Software; Validation; Visualization; Writing – original draft; Writing – review & editing. Kenji Leibnitz: Conceptualization; Investigation; Methodology; Resources; Software; Supervision; Writing – review & editing.

## FUNDING INFORMATION

Makoto Fukushima, Japan Society for the Promotion of Science (https://dx.doi.org/10.13039/501100001691), JSPS KAKENHI Grant Number: JP20H05066. Makoto Fukushima, Japan Society for the Promotion of Science (https://dx.doi.org/10.13039/501100001691), JSPS KAKENHI Grant Number: JP21K15610. Makoto Fukushima, The Uehara Memorial Foundation (https://dx.doi.org/10.13039/100008732).

## Supplementary Material









## TECHNICAL TERMS

Communication model: The framework that describes how neural elements interact and transmit signals within a network of structural brain connections.
Propagation strategy: The strategy for selecting a neighboring node to which a signal is transmitted on its way to the destination node.
Communication speed: The average number of signals that can be sent from source nodes to destination nodes per unit of time.
Information requirements: The amount of information about the network structure and node states used by signals to propagate toward their destination nodes.
Packet switching: A switching architecture in which each packet that is split from a signal is transmitted under a given propagation strategy.
Switching architecture: The mechanism by which a signal is transmitted across the network.
Message switching: A switching architecture in which an entire signal (message) is transmitted under a given propagation strategy.
Discrete-event simulation: A technique for modeling and simulating the operation of a system using discrete sequences of events in time.
