# Supplementary material for: Effects of packetization on communication dynamics in brain networks
Source: Netw Neurosci. 2024 Jul 1;8(2):418–36. doi: 10.1162/netn_a_00360 (PMC11142457; doi:10.1162/netn_a_00360)
Supplement: Supplementary file 1 [file netn-8-2-418-s001.pdf]

Supporting Information for

Effects of packetization on communication dynamics in brain networks

Makoto Fukushima[*], Kenji Leibnitz

[*]Corresponding author at Graduate School of Advanced Science and Engineering, Hiroshima University, Hiroshima, Japan

Email address: mfukushima@hiroshima-u.ac.jp

List of Supporting Information Files

- **File S1**. This file, containing the Supplementary Results and Supplementary Figures sections.
- **File S2**. Tables that display statistical details of the completion time comparisons.
- **File S3**. Source data of all figures.
- **File S4**. Code for generating all simulation results.

Supplementary Results

Comparison of transmission completion time with different simulation settings

To confirm our findings in the completion time comparisons (Figures 2B and 3B) in the Results section, we examined whether these results were robust against having the following changes applied to the default simulation setting of message switching and packet switching.

i) No buffer size limit

The limit on the buffer size in the default setting was removed. Figure S1 shows that the results were essentially the same as those obtained with the buffer size limit.

ii) Decrease/increase of the number of packets in a message

The number of packets n in a message was changed from five in the default setting to three and ten. While the different values of n changed the completion time of packet switching, the effects of packetization on the completion time (longer under RW and bRW with low control parameter c; shorter under iRW and bRW with intermediate c; unchanged under SP and bRW with high c) remained the same when c was decreased or increased (Figures S2 and S3).

iii) Messages of random lengths

The length of each message was varied from the default of five packets to a random integer number of packets sampled from the discrete uniform distribution ranging from two to eight with a mean of five. Unlike **ii)**, each message was composed of a different number of packets, where the service rate of each packet was 0.1 and that of each message was 0.1 divided by the number of packets in the corresponding message. The buffer can store messages or packets up to a total length equivalent to 100 packets. Figure S4 demonstrates that the results of the completion time comparisons were essentially the same as those obtained with the default setting. The completion times of packet switching under RW and bRW with low c (0.01 and 0.1) were reduced by having a distribution of message lengths, while they remained much longer than those of message switching.

iv) Decrease/increase of the arrival rate

The arrival rate λ was changed from 0.01 in the default setting to 0.005 (less frequent signal generation) and 0.02 (more frequent signal generation), which are the minimum and the maximum arrival rates used in Mišić, Sporns, & McIntosh (2014). As in this previous study, we kept the service rate μ unchanged because the system dynamics were dependent on the ratio λ/μ. Figure S5 shows that the effects of packetization

on the completion time also held with $\lambda = 0.005$. When $\lambda = 0.02$, packetization conversely increased the completion time under $\mathrm{iRW_a}$ (Figure S6A). However, the conclusion about the effects of packetization still held since the communication speed of $\mathrm{iRW_a}$ was much slower than that of the other versions of iRW in this case, and therefore $\mathrm{iRW_a}$ was no longer a strategy of balancing communication speed and information requirements at $\lambda = 0.02$. In bRW, while the range of c where packetization reduced the completion time became lower when $\lambda = 0.005$ (Figure S5B) and higher when $\lambda = 0.02$ (Figure S6B), the overall trend of the completion time difference between message switching and packet switching remained the same.

v) No packet overtaking

The simulation setting was changed to prevent packets from overtaking other packets from the same message. This was realized by first replacing the default assumption, where each packet can individually take its own route, with the assumption, where all packets in the same message follow the path of their foremost packet. Then, the default last-in-first-out queueing system, in which packets can be overtaken, was replaced with a first-in-first-out system. With this change, the oldest message or packet in a node's buffer was to start its service first after the service of the previously occupying message or packet at that node was finished, and a new message or packet arriving at a node was removed when its buffer was fully occupied by others. We observed that these changes shortened the completion time of packet switching under slow propagation strategies (e.g., RW); however, the effects of packetization on the completion time persisted (Figure S7).

Properties of individual messages and packet sets

In this subsection, we investigate properties of individual messages and packet sets that were successfully transmitted to their destination nodes. In our simulation samples, there were 100×100 such messages and packet sets for each combination of propagation strategy and switching architecture.

Transit time

We first focused on the duration from when a message or packet set was generated at the source node to when that message or the last packet arrived at the destination node, which corresponds to the transit time in Mišić, Sporns, & McIntosh (2014). Figure S8 shows the distribution of the transit time of each message or packet set. Note that the distributions presented in this figure do not account for messages and packets that did not reach their destinations, which can have long transit times. It should also be mentioned that the short transit times in these distributions do not necessarily mean fast communication. If the completion time for transmitting 100 messages or packet sets is too long, there is a possibility that the vast majority of messages and packet sets are still in transit while only a few arrive at their destinations and their transit times shape the distribution.

For RW and bRW with $c = 0.1$ and 0.6, the distribution of the transit time has a longer tail with packet switching due to the longer completion time to transmit 100 messages with packet switching. In contrast, the transit time distribution has a heavier tail with message switching under iRW and bRW with $c = 0.7$ and 1, where packetization reduced the transmission completion time. The heavier tail with message switching was also observed in SP and bRW with $c = 2$, where there was no difference in the transmission completion times between message switching and packet switching. Compared to message switching, the peak of the distribution with packet switching was located in a bin with longer transit times for propagation strategies other than $\mathrm{iRW_d}$ and $\mathrm{iRW_{a+d}}$.

Coverage

Next, we computed the coverage metric, which is defined as the fraction of nodes visited by a given message or any packet in a given packet set. Figure S9 displays the distribution of the coverage metric of each message or packet set that reached its destination node. As expected, the coverage with packet switching was higher than the coverage with message switching. In RW, the highest peak of the coverage distribution was located around 0.5 for packet switching, while the coverage was below 0.5 in most cases for message switching. The coverage profile for iRW depended on its version. Coverage for $\mathrm{iRW_a}$ was even higher than for RW, while coverage for $\mathrm{iRW_d}$ and $\mathrm{iRW_{a+d}}$ was low overall. The lowest coverage was observed for SP. The distribution of coverage for bRW changed continuously over the spectrum of c.

Supplementary Figures

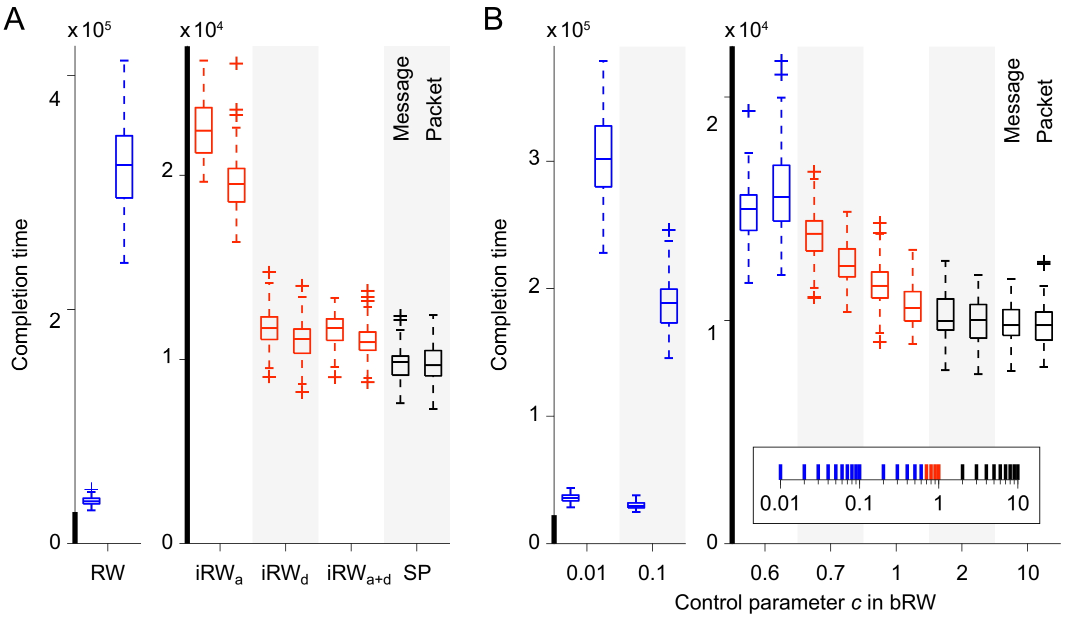

Figure S1. Completion time for transmitting 100 messages or packet sets in the simulations with no buffer size limit. (A) Completion time of RW, iRW, and SP. (B) Completion time of bRW. The boxplots are colored in the same manner as in Figures 2B and 3B.

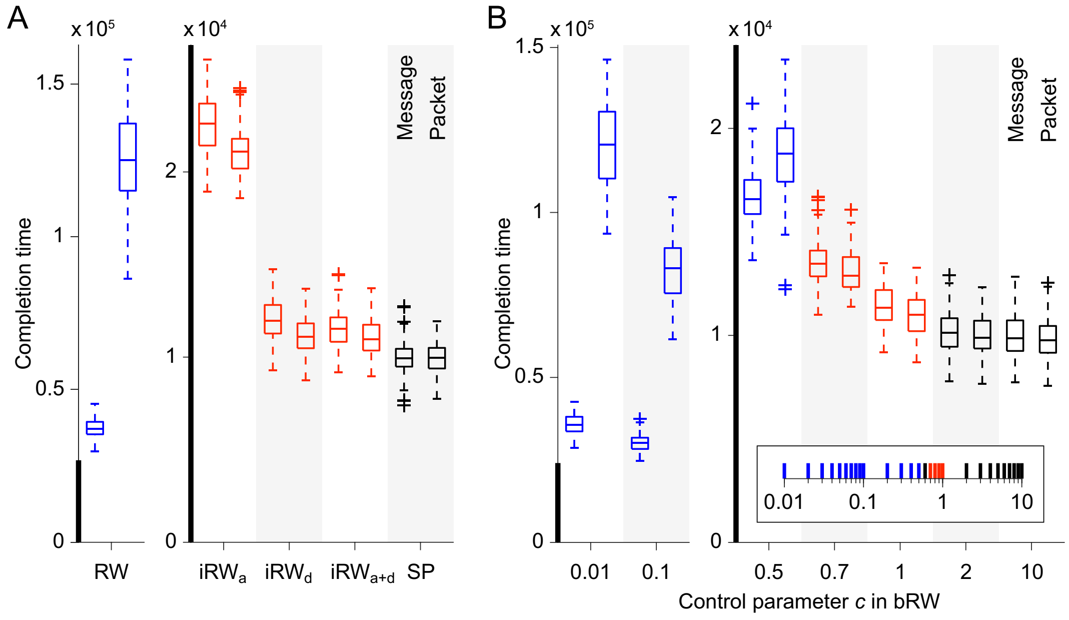

Figure S2. Completion time for transmitting 100 messages or packet sets in the simulations with the number of packets in a message $n = 3$. (A) Completion time of RW, iRW, and SP. (B) Completion time of bRW.

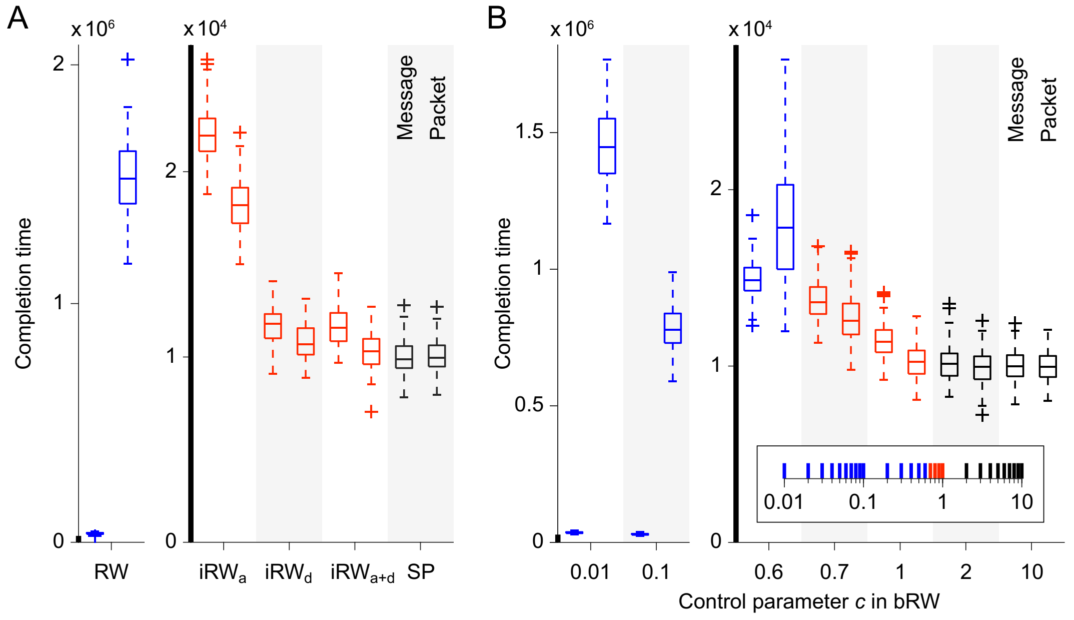

Figure S3. Completion time for transmitting 100 messages or packet sets in the simulations with the number of packets in a message $n = 10$. (A) Completion time of RW, iRW, and SP. (B) Completion time of bRW.

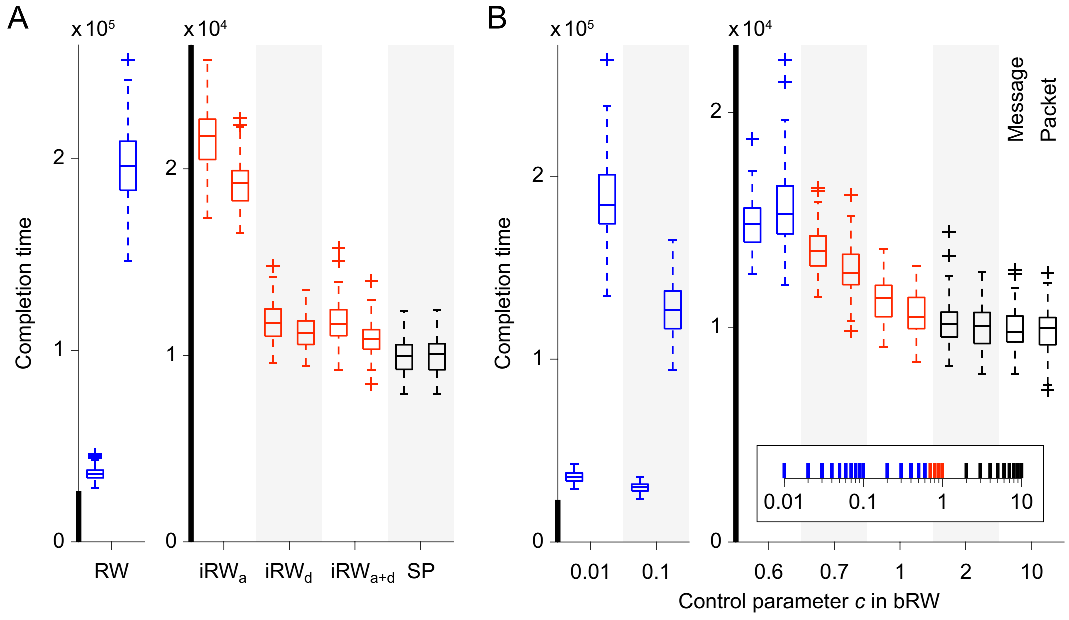

Figure S4. Completion time for transmitting 100 messages or packet sets in the simulations with messages of random lengths (2–8 packets). (A) Completion time of RW, iRW, and SP. (B) Completion time of bRW.

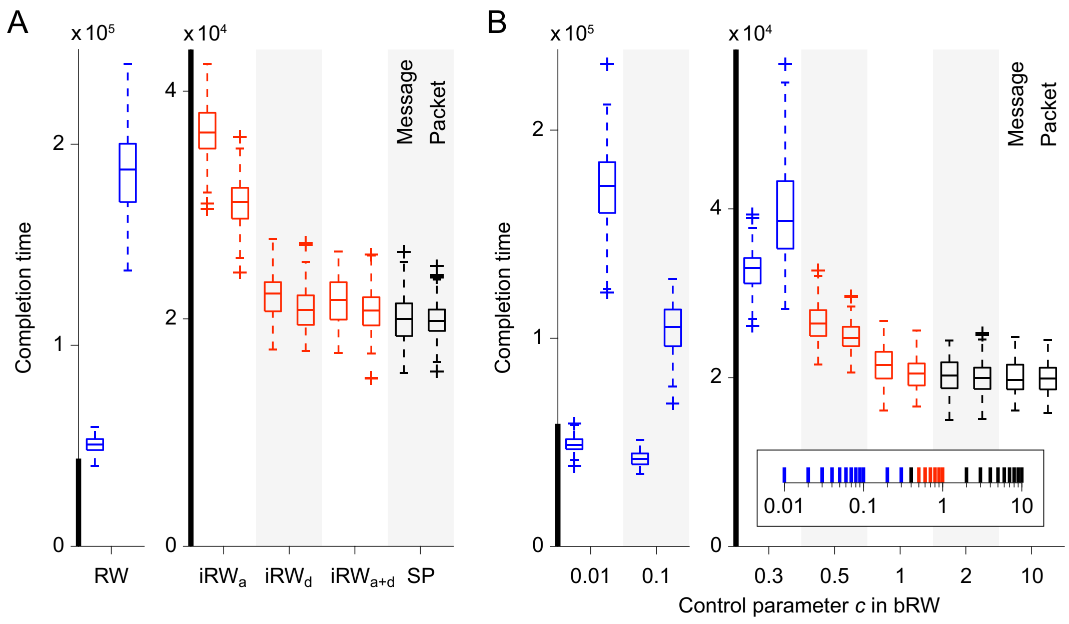

Figure S5. Completion time for transmitting 100 messages or packet sets in the simulations with the arrival rate $\lambda = 0.005$. (A) Completion time of RW, iRW, and SP. (B) Completion time of bRW.

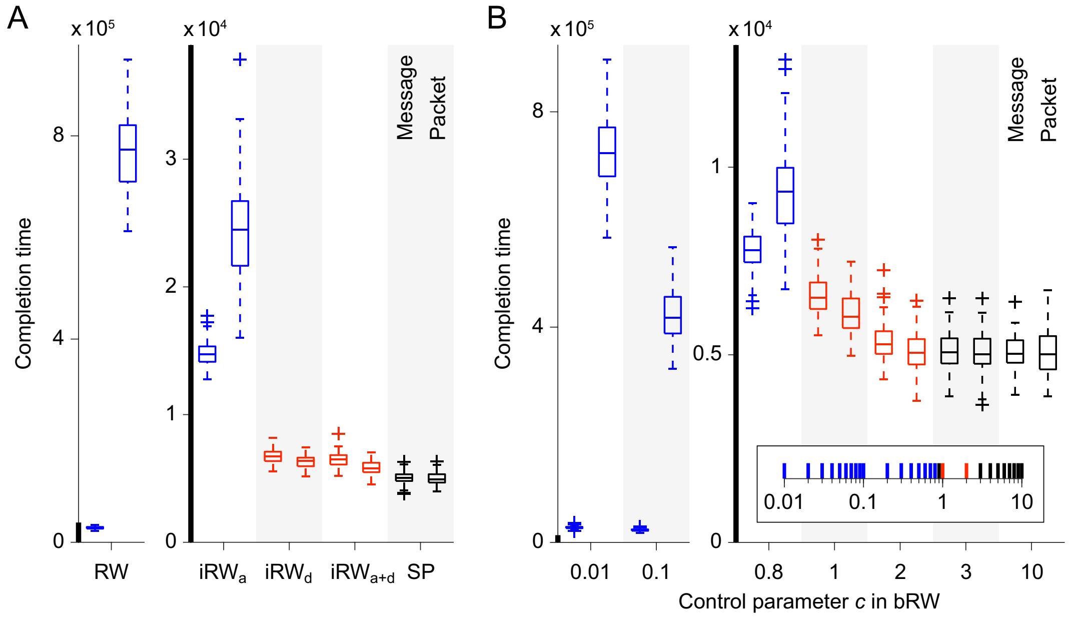

Figure S6. Completion time for transmitting 100 messages or packet sets in the simulations with the arrival rate $\lambda = 0.02$. (A) Completion time of RW, iRW, and SP. (B) Completion time of bRW.

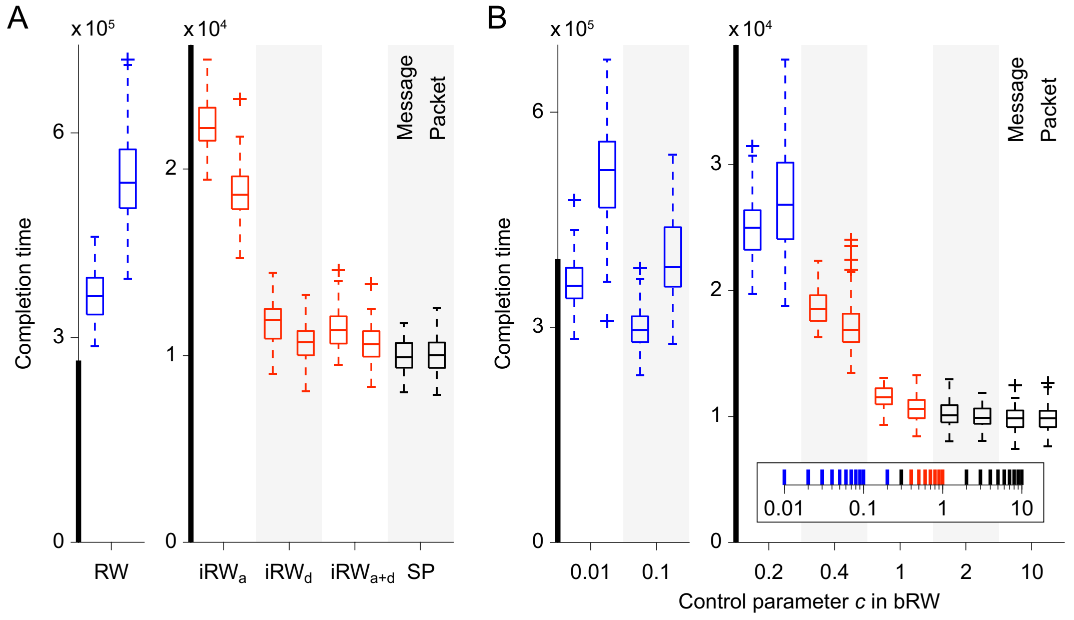

Figure S7. Completion time for transmitting 100 messages or packet sets in the simulations with no packet overtaking. (A) Completion time of RW, iRW, and SP. (B) Completion time of bRW.

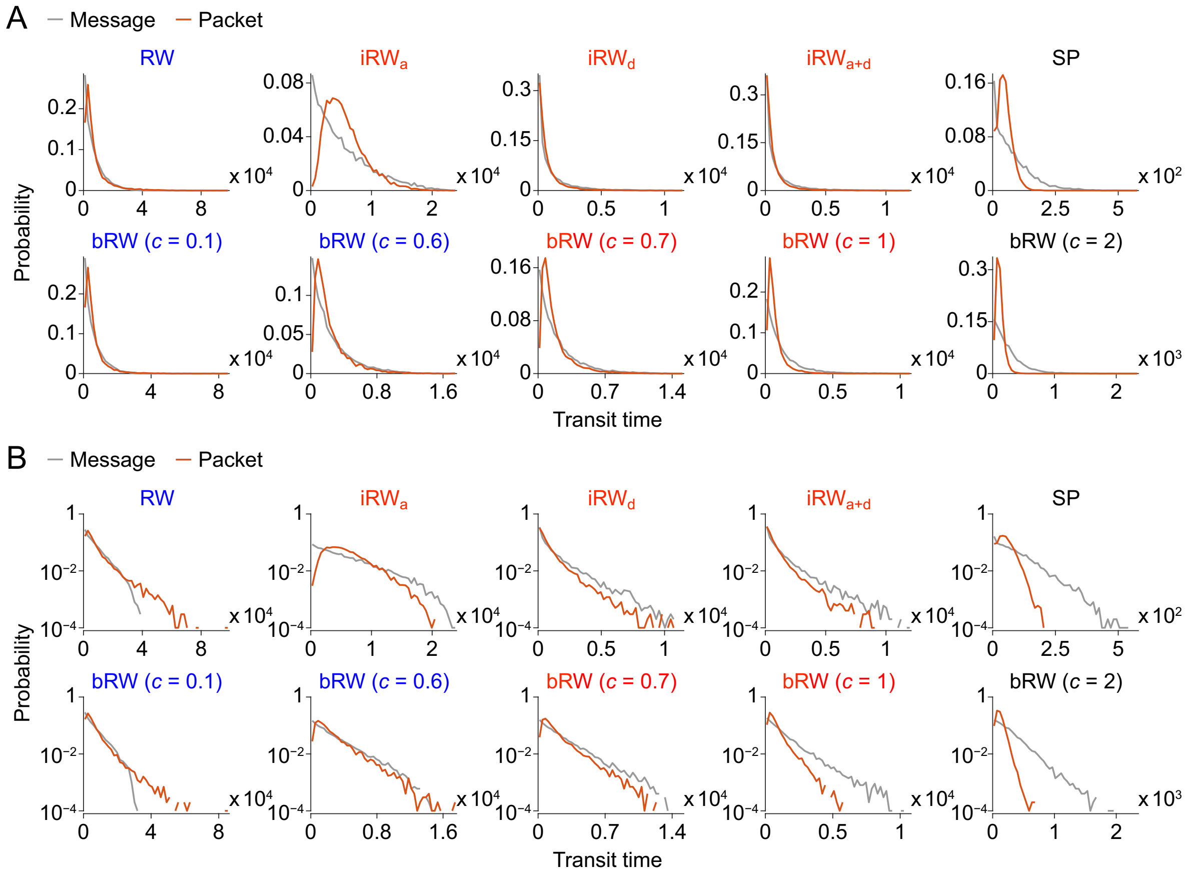

Figure S8. Distribution of the transit time of each message or packet set (A: linear scale, B: logarithmic scale). Each line graph of the histogram (the number of bins: 50) is drawn from the 100×100 messages or packet sets that were successfully transmitted to their destination nodes.

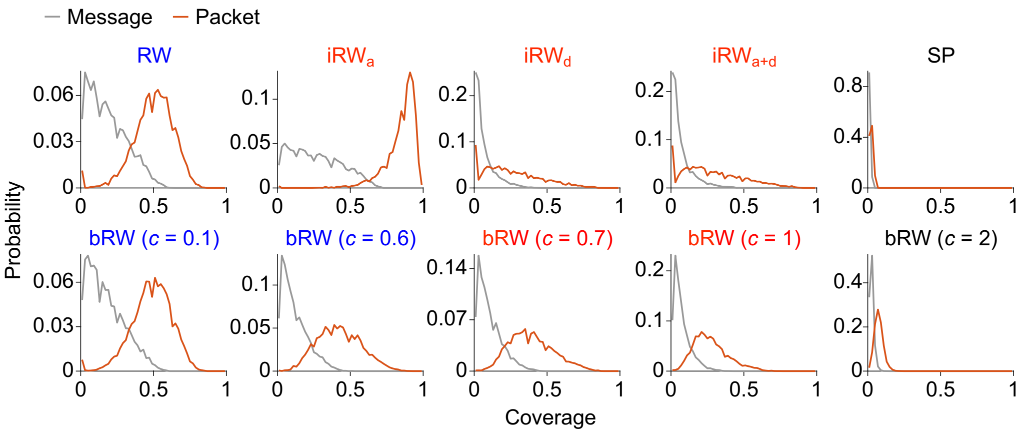

Figure S9. Distribution of the coverage metric of each message or packet set. Each line graph of the histogram (the number of bins: 50) is drawn from the 100×100 messages or packet sets that were successfully transmitted to their destination nodes.

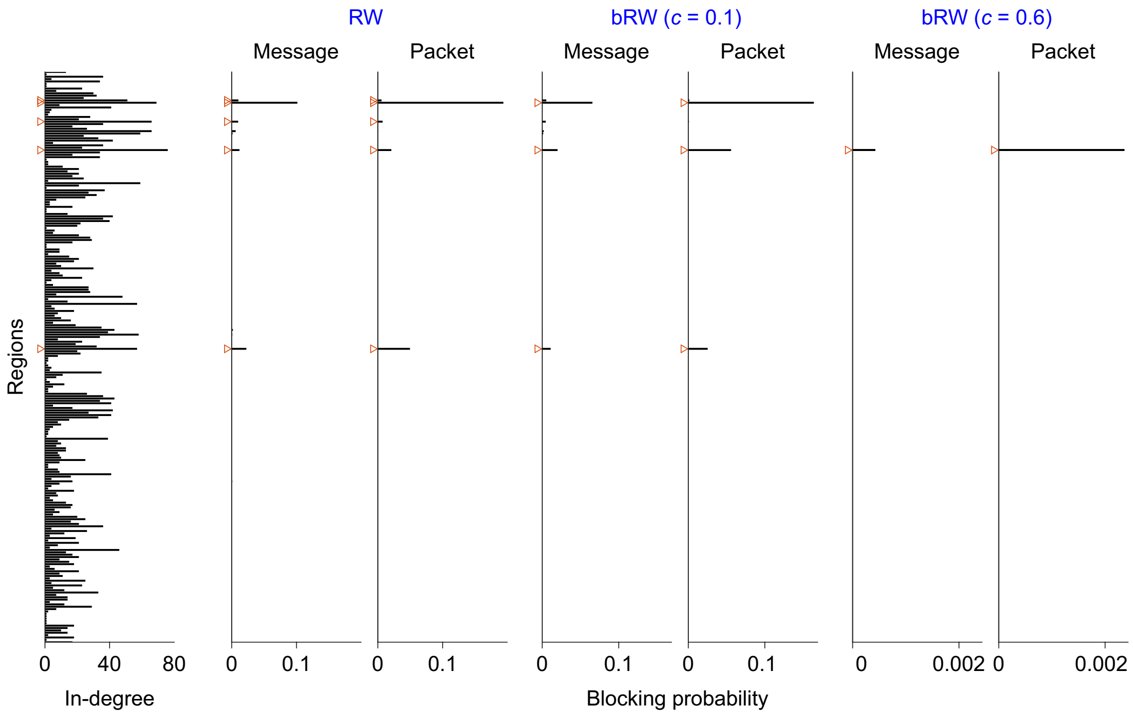

Figure S10. In-degree and blocking probability of nodes under RW and bRW with $c = 0.1$ and 0.6. The blocking probability was defined as the number of times that messages or packets were dropped from the buffer at a node divided by the total number of messages or packets arriving at that node. The triangles along the vertical axis indicate the locations of the bottleneck nodes under RW for in-degree and the nodes surrounded by the ellipses in Figure 4B for blocking probability.

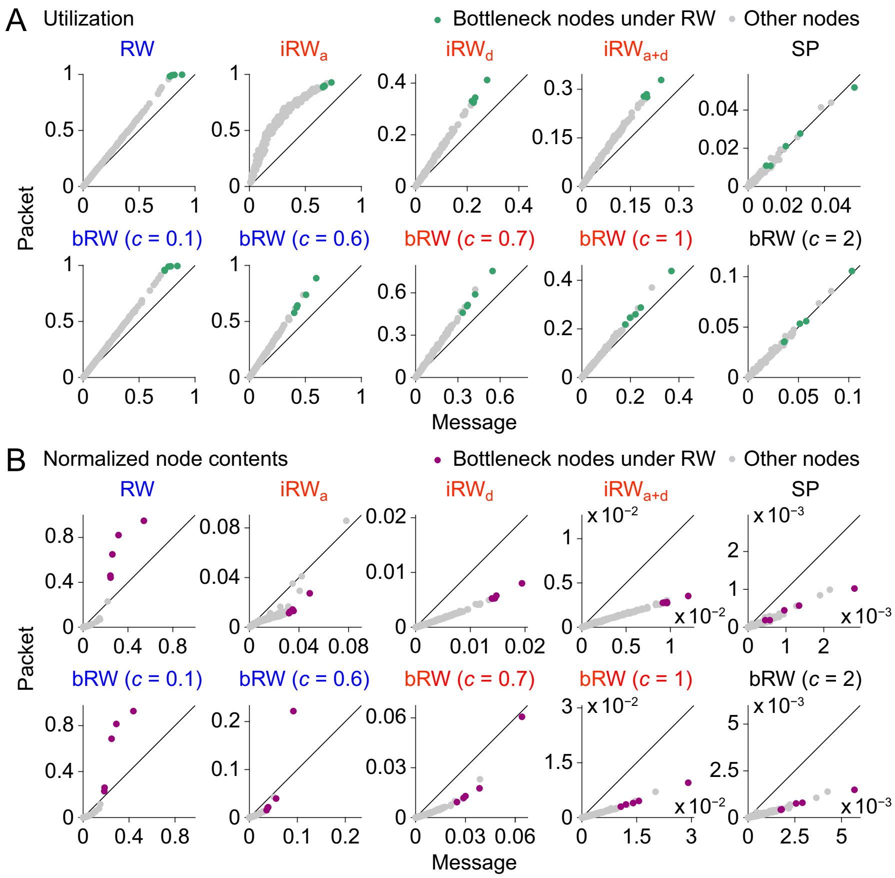

Figure S11. The scatter plots in Figure 4A and B in a different color scheme. The dots corresponding to the bottleneck nodes under RW are colored green in (A) (utilization) and purple in (B) (normalized node contents). The other dots are shown in gray.

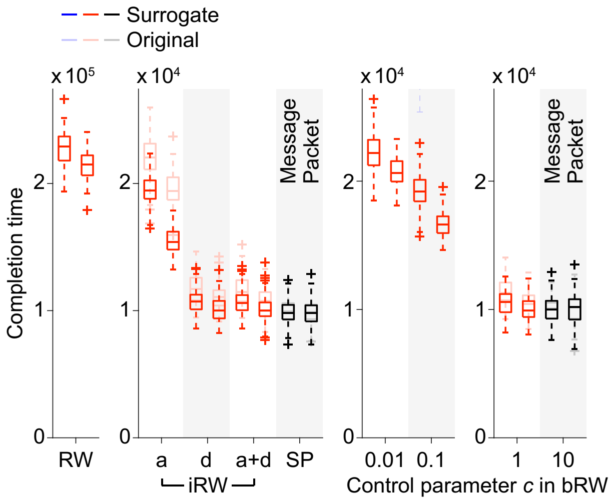

Figure S12. The boxplots zoomed in along the vertical axis for RW and bRW with $c = 0.1$ and 0.01 in Figure 5C. The boxplots for iRW, SP, and bRW with $c = 1$ and 10 are also shown with the same vertical axis range.
